# Differential effect of disease-associated *ST8SIA2* haplotype on cerebral white matter diffusion properties in schizophrenia and healthy controls

**DOI:** 10.1038/s41398-017-0052-z

**Published:** 2018-01-22

**Authors:** Janice M. Fullerton, Paul Klauser, Rhoshel K. Lenroot, Alex D. Shaw, Bronwyn Overs, Anna Heath, Murray J. Cairns, Joshua Atkins, Rodney Scott, The Australian Schizophrenia Research Bank, Peter R. Schofield, Cyndi Shannon Weickert, Christos Pantelis, Alex Fornito, Thomas J. Whitford, Thomas W. Weickert, Andrew Zalesky

**Affiliations:** 1 Neuroscience Research Australia, Randwick, Sydney, NSW Australia; 2 School of Medical Sciences, Faculty of Medicine, University of New South Wales, Sydney, NSW Australia; 3 Schizophrenia Research Institute, Sydney, NSW Australia; 4 Brain and Mental Health Laboratory, Monash Institute of Cognitive and Clinical Neurosciences, School of Psychological Sciences and Monash Biomedical Imaging, Monash University, Clayton, VIC Australia; 5 Melbourne Neuropsychiatry Centre, The University of Melbourne and Melbourne Health, Carlton South, VIC Australia; 6 School of Psychiatry, Faculty of Medicine, University of New South Wales, Sydney, NSW Australia; 7 School of Biomedical Sciences, University of Newcastle, Newcastle, NSW Australia; 8 School of Psychology, Faculty of Science, University of New South Wales, Sydney, NSW Australia

## Abstract

Brain white matter abnormalities are evident in individuals with schizophrenia, and also their first-degree relatives, suggesting that some alterations may relate to underlying genetic risk. The ST8 alpha-N-acetyl-neuraminide alpha-2,8-sialyltransferase 2 (*ST8SIA2)* gene, which encodes the alpha-2,8-sialyltransferase 8B enzyme that aids neuronal migration and synaptic plasticity, was previously implicated as a schizophrenia susceptibility gene. This study examined the extent to which specific haplotypes in *ST8SIA2* influence white matter microstructure using diffusion-weighted imaging of individuals with schizophrenia (*n* = 281) and healthy controls (*n* = 172), recruited across five Australian sites. Interactions between diagnostic status and the number of haplotype copies (0 or ≥1) were tested across all white matter voxels with cluster-based statistics. Fractional anisotropy (FA) in the right parietal lobe was found to show a significant interaction between diagnosis and *ST8SIA2* protective haplotype (*p* < 0.05, family-wise error rate (FWER) cluster-corrected). The protective haplotype was associated with increased FA in controls, but this effect was reversed in people with schizophrenia. White matter fiber tracking revealed that the region-of-interest was traversed by portions of the superior longitudinal fasciculus, corona radiata, and posterior limb of internal capsule. Post hoc analysis revealed that reduced FA in this regional juncture correlated with reduced IQ in people with schizophrenia. The *ST8SIA2* risk haplotype copy number did not show any differential effects on white matter. This study provides a link between a common disease-associated haplotype and specific changes in white matter microstructure, which may relate to resilience or risk for mental illness, providing further compelling evidence for involvement of *ST8SIA2* in the pathophysiology of schizophrenia.

## Introduction

Schizophrenia and bipolar disorder are severe psychiatric conditions, comprising constellations of overlapping clinical symptoms and shared genetic risk^1,2^. There is evidence of both regionally specific and widespread white matter abnormalities in bipolar disorder^3,4^ and schizophrenia^5,6,7^ compared to healthy controls, although there is some inconsistency among reports of specific white matter tracts implicated in individual studies. These inconsistencies are likely owing to a combination of methodological differences across studies, variation in power to detect group differences, and heterogeneity at the clinical, demographic, and genetic levels within subject groups.^8^ Indeed, some cerebral abnormalities may relate specifically to underlying genetic risk (rather than to disease state), as evidenced by white matter abnormalities observed in first-degree relatives of patients^9,10^, although non-genetic familial risk factors may also contribute.

A growing number of genes that carry variation associated with altered risk of disease have been implicated in schizophrenia^11^. Understanding how established genetic risk variants influence white matter tract formation in healthy and affected brains is pivotal in dissecting out sources of heterogeneity in neuroimaging studies focused on diagnostic group differences, but also in understanding the complex underlying neurobiology of those disorders.

DNA variations within the ST8 alpha-N-acetyl-neuraminide alpha-2,8-sialyltransferase 2 gene *(ST8SIA2*) have previously shown association with a number of major psychiatric conditions, including bipolar disorder^12^, schizophrenia^12–14^, and autism^15^. In addition, loss-of-function mutations affecting *ST8SIA2* have been identified in individuals with schizophrenia^16^, and autism spectrum disorder with epilepsy^17^.

*ST8SIA2* encodes the alpha-2,8-sialyltransferase 8B enzyme, responsible for the post-translational addition of polysialic acid (PSA) onto proteins, principally the neuronal cell adhesion molecule (*NCAM1*) during early brain development^18,19^, enabling neuronal migration, dendrite formation, axon targeting, and synaptic plasticity^20^. Observations of altered PSA-NCAM in brains of patients with schizophrenia, bipolar disorder, major depression, and drug-refractory temporal lobe epilepsy indicate a functional dysregulation of the glycosylation process in mental illness^21–26^. This notion is supported by pharmacological data in rats, in which expression of PSA-NCAM is modulated by treatment with common antipsychotics^27–29^.

Mouse knockout studies show evidence of cerebral changes in animals developing without glycosylated NCAM, including the size of the anterior commissure and midline-crossing fibers, ventricular dilatations, size reductions of the internal capsule, and disorganized pattern of fibers^30,31^. In addition, *st8sia2*-deficient mice (*st8sia2*^−/−^) exhibit schizophrenia-like behaviors, including cognitive dysfunction, deficits in prepulse inhibition, and increased sensitivity to amphetamine-induced locomotion^31^. These animal models suggest that cerebral changes may also be observed in humans with specific *ST8SIA2* risk alleles, albeit with more subtle abnormalities relating to less severe genetic defects, which may include alterations in the organization of white matter brain connectivity.

Therefore, in the current study, we aimed to examine the extent to which *ST8SIA2* influences white matter structure in a human cohort, comprising both people with schizophrenia and healthy controls. As no single common functional *ST8SIA2* mutation has been identified^16,32^, we focused on a specific 54-kb linkage disequilibrium block encompassing the promoter to intron 2, which contained two common haplotypes previously identified as carrying “risk” or “protective” alleles^12^. Individuals were grouped both according to diagnosis and the presence of either the risk or protective haplotype. We then tested across all white matter voxels whether fractional anisotropy (FA)—a measure of white matter microstructure—was modulated by the interaction between haplotype and diagnosis. The presence of a significant interaction was tested across all white matter using an unbiased, data-driven method, thereby investigating the differential influence of *ST8SIA2* haplotypes on white matter structure in healthy controls and in people with schizophrenia.

## Materials and methods

### Participants

Participants were 18–65 years of age and included patients with established schizophrenia (*n* = 281) and healthy controls (*n* = 172) recruited from five sites in Australia under the auspices of the Australian Schizophrenia Research Bank^33^ (Ethics Committee approval by University of New South Wales HREC/08 and Hunter New England Human Research Ethics Committee HNE/438). All participants provided written informed consent for the analysis of their data. A diagnosis of schizophrenia or schizoaffective disorder was determined using DSM-IV diagnostic criteria^34,35^. Current Intelligence Quotient (IQ) estimates were obtained using the Wechsler Abbreviated Scale of Intelligence^36^.

Exclusion criteria were as previously described^33,37^, and included a history of organic brain disorders, brain injury (followed by amnesia for >24 h), movement disorders, a current diagnosis of drug or alcohol dependence, or electroconvulsive therapy in the past 6 months. Healthy controls were also excluded if they had a familial or personal history of psychosis or bipolar I disorder.

Ethnicity was determined by a combination of: (1) self-report, based on grandparental country of birth; (2) genotype-derived principle components analysis, where GWAS data were available^37^ (*n* = 333, 68% of sample; Supplementary Information), and was largely European (88.1%) or mixed-European (7.0%; Supplementary Table S1).

### Genotyping

**Fig. 1:**
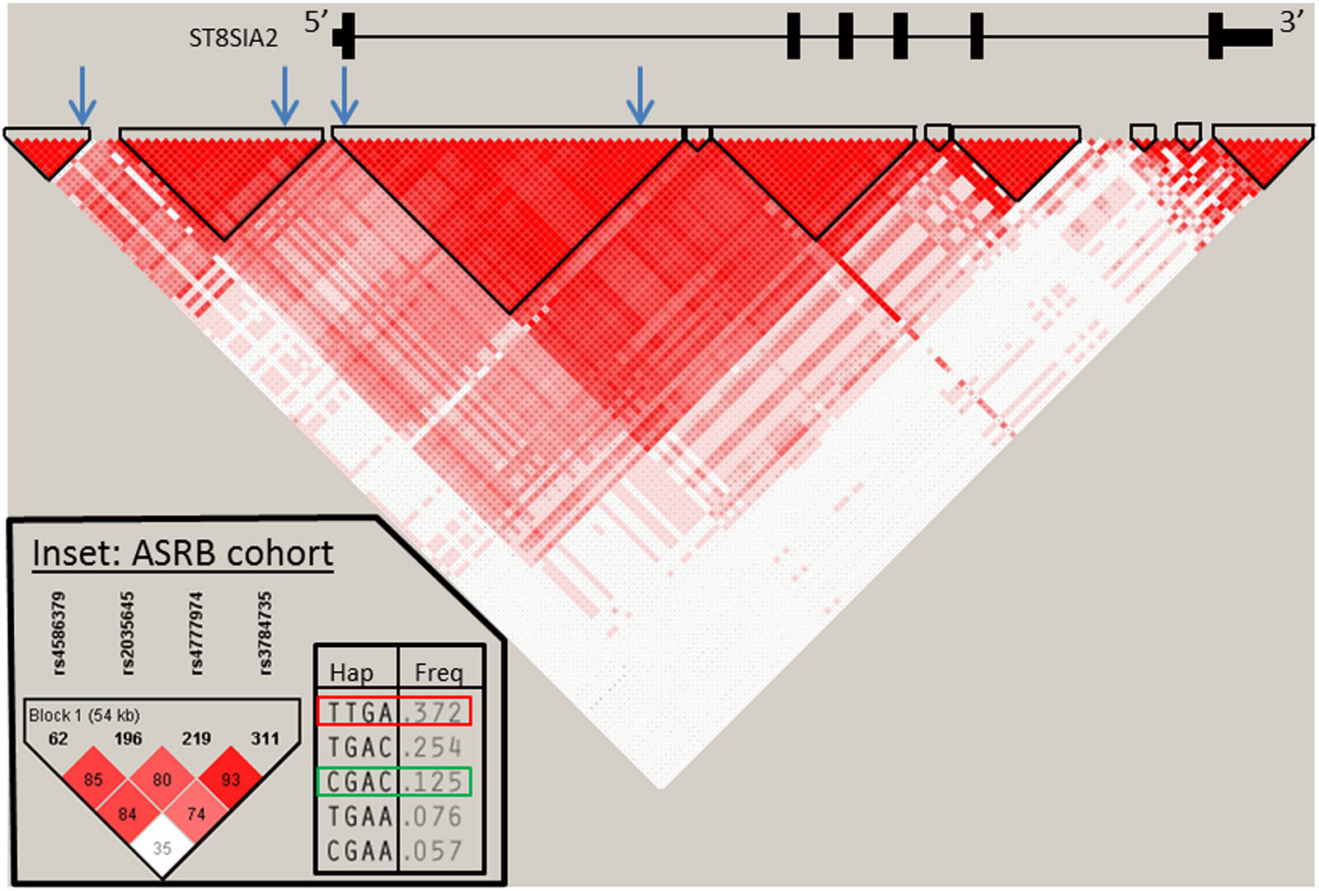
Position of genotyped SNPs within the *ST8SIA2* gene, and phased haplotypes The haplotype block structure across the entire ST8SIA2 gene (chr15:92,910–92,995 Mb; hg18) is shown, determined from 174 European (CEU, GBR) individuals from phase 1 of the 1000 Genomes project (MAF > 0.2). *ST8SIA2* gene structure is shown above, with black bars indicating exon positions and the promoter region lying in the 5’ direction. Relative LD strength is indicated by strength of red coloring, whereby dark red indicates high LD (*D*’ > 0.8). Blue arrows indicate position of SNPs genotyped in the current study (rs4586379, rs2035645, rs4777974, and rs3784735). The main haplotypes (frequency > 0.05) defined across the four genotyped SNPs are shown in the inset, alongside their observed frequencies (freq). The TTGA and CGAC haplotypes (indicated in the red and green boxes, respectively) correspond to the previously identified “risk” and “protective” haplotypes, as reported in McAuley et al.^12^

Putative risk and protective haplotypes^12^ were defined by four single nucleotide polymorphisms (SNPs) located toward the 5’ end of *ST8SIA2* (Fig. 1). Lymphocyte-derived genomic DNA underwent PCR amplification using Taqman probes to generate genotypes for each SNP, and haplotypes were phased using PLINK^38^ (Supplementary Information).

For seven subjects where phasing was deemed unreliable (posterior probability <0.75), imputed data from Infinium Human 610 K BeadChips^37^ were used (Supplementary Information), for whom the average posterior probability-phased haplotypes was 0.843. Haplotype association with diagnosis was performed in PLINK^38^ using *--hap-logistic*, including ethnicity as a covariate.

### Image acquisition

Diffusion-weighted magnetic resonance images (DWI) were acquired in each participant with a Siemens Avanto 1.5-Tesla system (Siemens, Erlangen, Germany) across five sites in Australia, with the same acquisition sequence used across all sites. Sixty-four gradient-weighted volumes distributed on the half-sphere were acquired using a spin-echo EPI sequence as follows: *b*-value = 1000 s/mm^2^; 65 consecutive axial slices (thickness 2.4 mm); 104 × 104 image matrix with an in-plane voxel resolution of 2.4 × 2.4 mm; field of view = 25 × 25 cm; repetition time = 8.4/8.5 s; echo time = 88 ms; flip angle = 90 degrees.

### Image preprocessing

DWI images were corrected for EPI distortions and head movement with affine registration to the non-diffusion-weighted volume in FSL 5.0.7 (http://fsl.fmrib.ox.ac.uk/fsl/fslwiki/). Gradient tables were rotated to correct for head movement using MATLAB v2011a (MathWorks, Natick, MA, USA). Diffusion tensors were then fitted to each voxel using least squares estimation, enabling generation of a FA image for each individual in MRtrix 0.2.12 (http://www.nitrc.org/projects/mrtrix/). FA is a voxel-wise measure that reflects the degree to which water diffusion is restricted to particular directions. FA is influenced by microstructural properties and organization of white matter fibers including axons, their myelin sheath, and the surrounding extracellular matrix^39^. Each FA image was normalized to Montreal Neurological Institute (MNI) standard space using a nonlinear registration procedure (FLIRT and FNIRT with default parameters as implemented in FSL 5.0.7). Quality control included careful manual inspection of each FA image for gross abnormalities and/or registration failure. Streamlines were seeded throughout all of white matter and propagated using a deterministic white matter fiber tracking algorithm in MRtrix 0.2.12 (Supplementary Information).

### Statistical analysis

A two-way analysis of variance was used to test for interactions between diagnostic status (patient or control) and the number of haplotype copies (0, 1 or 2) at each white matter voxel. FA was the dependent variable, and independent variables were the main effects of diagnosis and haplotype, the interaction between these two main effects, and the nuisance covariates of scanning site, age, and gender. The main effect of haplotype was modeled in two ways: (1) additively, such that two haplotype copies were assumed to have double the effect of one haplotype copy over no copies; and (2) dominantly, pooling together individuals with one or two haplotype copies to form a single group. The latter approach accounted for the rareness of individuals with two haplotype copies. This model was independently fitted at all white matter voxels. Whole-brain correction for multiple comparisons (across all white matter voxels) was conducted using Randomise (FSL 5.0.7), a non-parametric cluster-size-based procedure^40,41^, which avoids inflation of false-positives^42^. A primary t-statistic threshold of 2.5 was used. Corrected *p* values for each cluster were calculated based on 10,000 permutations, and two-sided *p* values < 0.05 were considered significant. Post hoc analysis methods are described in Supplementary Information.

## Results

The four SNPs defining the risk and protective haplotypes previously identified^12^ (Fig. 1), each had a genotyping rate of >98%. All SNPs passed tests for Hardy–Weinberg equilibrium in control subjects. After haplotype phasing, 39 subjects (20 cases, 19 controls) were excluded due to low posterior probability of phased haplotypes, given the observed genotypes (<0.70). In total, 453 subjects (281 cases, 172 controls) had both high-quality FA images and confidently phased haplotypes (average posterior probability was 0.961; Table 1).

**Table 1:** Breakdown of haplotype status by diagnostic group

	TTGA “risk” haplotype		CGAC “protective” haplotype		Total
Number of haplotype copies	0	1+ (2)	0	1+ (2)	
Case	115	166 (36)	207	74 (9)	281
Control	64	108 (17)	127	45 (5)	172
Total	179	274 (53)	334	119 (14)	453

Subjects with 0 copies of the “risk” or “protective” haplotypes have one of seven other haplotypes on both chromosomes, namely, TGAC, TGAA, TTAC, CGAA, CTGA, TGGA, or TTGC. Subjects with 1+ copies represent subjects with one or more copies of the relevant haplotype, and homozygotes for each haplotype (2 copies) shown in parentheses. Subjects with one copy of the relevant haplotype may have any other haplotype on the alternate chromosome, including subjects who have one copy of both risk and protective haplotypes (*n* = 26 cases, 21 controls)

There was no significant difference in frequency of common haplotypes (frequency > 0.05) in controls vs. patients (omnibus Wald T = 0.122, df = 3, *p* = 0.989), nor was there altered frequency of the specific risk (*χ*^2^ = 0.616, df = 1, *p* = 0.432) or protective haplotypes (*χ*^2^ = 0.002, df = 1, *p* = 0.967) with diagnostic group. There were no significant differences in age, gender, handedness, or IQ in carriers vs. non-carriers of either risk or protective haplotypes in either diagnostic group. The schizophrenia group had significantly more males, a lower median IQ score (Table 2 and Supplementary Figure S1), and widespread reductions of FA compared to controls^6^.

**Table 2:** Demographics, clinical variables, and protective haplotype (CGAC) carrier status

	Controls				Patients				Controls vs. Patients
CGAC “protective” haplotype copies	All (*n* = 172)	0 copies (*n* = 127)	1 + copies (*n* = 45)	Carrier vs. non-carrier	All (*n* = 281)	0 copies (*n* = 207)	1 + copies (*n* = 74)	Carrier vs. non-carrier	*X*^*2*^ = 0.002 (*p* = 0.967)
Age	41.5 (18–64)	42	40	*U* = 2762	38	37	38.5	*U* = 7452	*U* = 22,208
	(18-64)	(18–64)	(18–62)	(*p* = 0.739)	(20–65)	(20–65)	(20–63)	(*p* = 0.729)	(*p* = 0.147)
Sex (males; females)	87; 85	69; 58	18; 27	*X*^2^ = 2.73	196; 85	147; 60	49; 25	*X*^2* = *^0.595	***X*** ^***2***^ ** = 16.72**
				(*p* = 0.098)				(*p* = 0.44)	(*p* **= 4.3 × 10**^**−5**^**)**
Handedness	90	90	90	*U* = 2729	100	100	100	*U* = 7626	*U* = 21,856
				(*p* = 0.635)				(*p* = 0.952)	(*p* = 0.066)
WASI	119	118	121	*U = *2475	104	105	103.5	*U = *7051	***U*** ** = 11,645**
	(80–138)	(80–138)	(88–134)	(*p* = 0.182)	(58–133)	(58–132)	(63–133)	(*p* = 0.311)	(*p* **= 2.0 × 10**^**−20**^**)**
Diagnostic (SCZ; SAD; SAB)		—	—	—		176; 18; 13	63; 6; 5	*X*^*2*^ = 0.041 (*p* = 0.979)	—

DSM-IV diagnostics (*SCZ* schizophrenia; *SAD* schizoaffective disorder of depressive type, *SAB* schizoaffective disorder bipolar type); handedness as measured by Edinburgh Handedness Scale, a continuous laterality quotient scaled from −100 to +100, where negative values indicate propensity for left-handedness and ±100 indicates unilaterality; *WASI* Wechsler abbreviated scale of intelligence; “Carrier” refers to carrier of CGAC “protective” haplotype; if not otherwise specified, the values represent the median and the range is given in brackets; statistically significant differences (*p* < 0.05) are indicated in bold

**Fig. 2:**
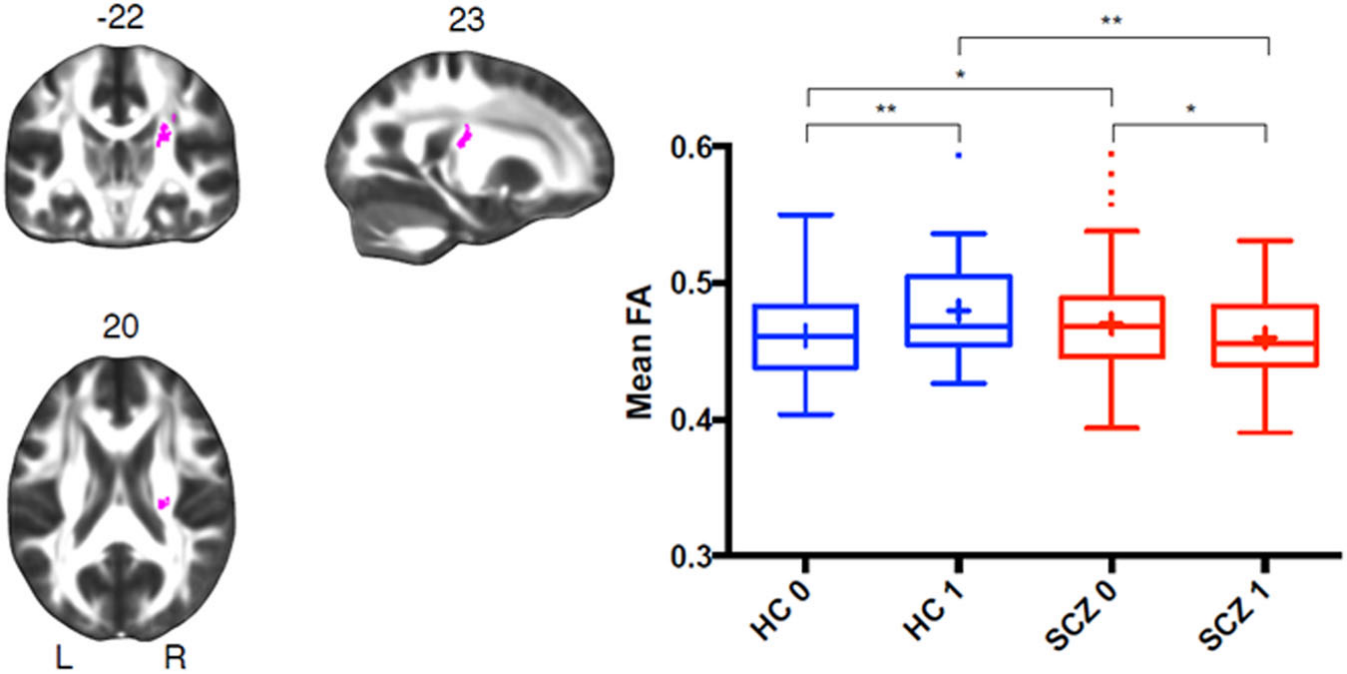
Differential effect of ST8SIA2-protective haplotype on fractional anisotropy in healthy controls and subjects with schizophrenia The pink cluster (804 voxels) located on the right corona radiata and the superior longitudinal fasciculus represents voxels showing a significant interaction between *ST8SIA2* protective haplotype and diagnosis of schizophrenia on fractional anisotropy. MNI coordinates are given on the top of each slice. The cluster is significant at the whole brain level (corrected *p* *= *0.04, family-wise error rate-corrected). The Tukey Box-And-Whiskers plot represents the distribution of the mean FA values extracted from the pink cluster, with whiskers representing 1.5 interquartile range. Healthy controls (HC, *n* = 172, blue) and subjects with schizophrenia (SCZ, *n* = 281, red) carrying one or two copies (1) or no copy (0) of the *ST8SIA2*-protective haplotype are shown. Results of the post hoc tests are indicated: *p *< *0.05; **p *< *0.01

Individuals (*n* = 453) were grouped according to diagnosis, and the presence of either the risk or protective haplotype. No significant main effects of risk or protective haplotype on FA were observed (*p* > 0.5). In two separate analyses for each haplotype, we then tested whether FA was modulated by the interaction between haplotype and diagnosis. The number of copies of the *ST8SIA2* risk haplotype (TTGA) did not show any differential effects on white matter with diagnosis. However, the *ST8SIA2* protective haplotype (CGAC) was found to have a differential effect on right hemisphere parietal white matter in patients with schizophrenia, and was significant for both the pooled and additive modeling approach (whole brain-corrected *p* < 0.05, 804 voxels; Fig. 2 and Supplementary Figure S2, respectively). The interaction effect was stronger when subjects of Asian or unknown ancestry were excluded (*n* = 401; dominant model *p* = 0.0052, 2401 voxels; Supplementary Figure S3).

**Fig. 3:**
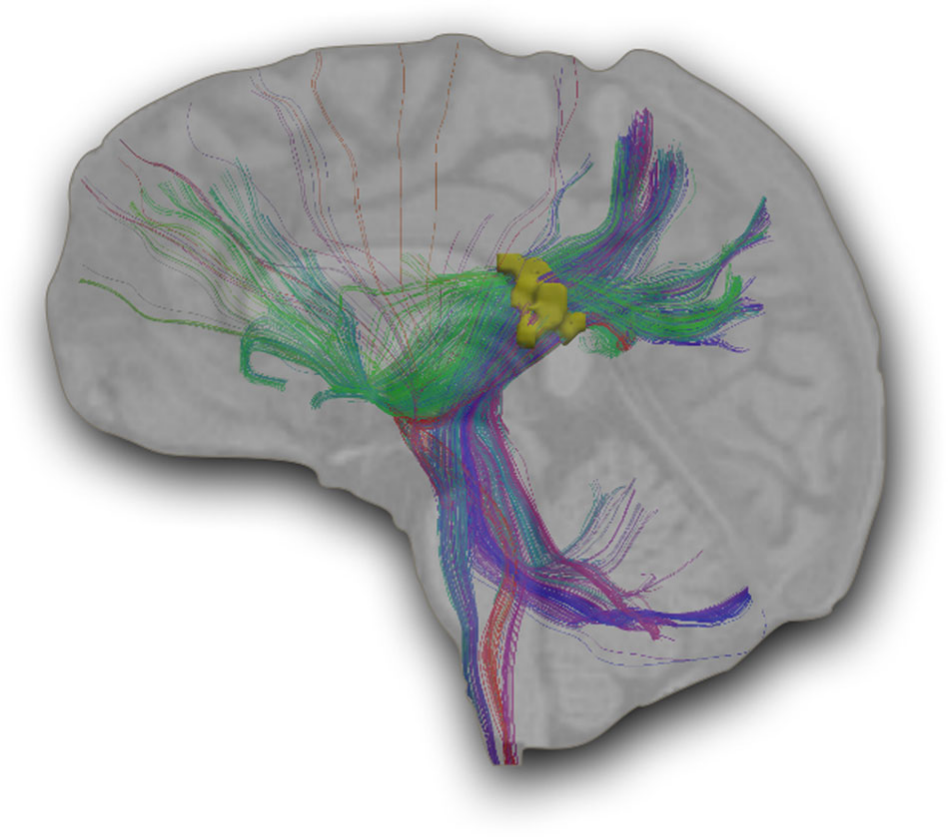
Involvement of the superior longitudinal fasciclus and corona radiata Tractography was performed for a representative control to delineate the white matter fiber bundles traversing the region associated with the significant protective haplotype × diagnostic group interaction (yellow), which was derived from all subjects (*n* = 453). The dominant fiber bundles are the superior longitudinal fasciculus (horizontal fibers, mostly green) and the corona radiata (vertical fibers, mostly blue)

The anatomical location of the significant right hemisphere interaction extended between the right superior longitudinal fasciculus (SLF) and the posterior limb of the internal capsule, with a peak *t-*statistic voxel located in the superior corona radiata (MNI mm: 23, −22, 20; Fig. 2, left panel). The protective haplotype was associated with increased FA in this region in controls, but decreased FA in the patient group. This was confirmed with post hoc testing using mean FA values averaged across all voxels in the region showing the significant interaction effect (Fig. 2, right panel). The cluster was slightly more significant (*p* = 0.036, 876 voxels) when five patients with IQ > 2 S.D. outside the group mean (mean = 103.3, S.D. = 15.7) were excluded (data not shown). The mean FA was significantly greater in patients compared to controls in individuals with no copies of the protective haplotype. Conversely, the mean FA was significantly greater in controls compared to patients with one or more copies of the protective haplotype. To identify the axonal fiber bundles associated with the interaction effect, white matter fiber tracking was performed by initiating streamlines from all white matter voxels, but retaining only those passing through the region. The set of 1000 reconstructed streamlines that intersected the cluster (Fig. 3) shows that the region of the significant interaction effect is traversed by portions of both corticocortical white matter tracts (i.e., SLF) and corticosubcortical white matter tracts (i.e., corona radiata and posterior limb of internal capsule). In the ethnicity-restricted analysis (excluding 52 subjects of Asian or unknown ancestry), the significant interaction region extended more caudally, descending from the SLF and corona radiata to the cerebral peduncles (midbrain), passing through the posterior limb of the internal capsule (Supplementary Figure S4). The interaction effect, therefore, appears to be associated with a regional juncture of multiple fiber bundles, including both association and projection fiber bundles.

Finally, we conducted a post hoc examination of the relationship between the mean FA within the region-of-interest (FA-ROI) and IQ, as cognitive impairment is a core component of schizophrenia that may be causal^43^. Although age was included as a covariate in primary analyses, age has been shown to significantly affect FA^44^; thus, we explicitly examined effects of age on FA-ROI. Regression analysis revealed a significant positive correlation between FA-ROI and IQ in Caucasian people with schizophrenia (*t* = 2.276; df = 7,227; *p* = 0.024), but no significant relationship with IQ in controls (*t* = 1.633; df = 7,122; *p* = 0.105). When haplotype was included in the model, both IQ and haplotype were significant predictors on FA-ROI in people with schizophrenia (*t* = 2.197; df = 8,227; *p* = 0.029, and *t* = 2.144; df = 8,227; *p* = 0.033, respectively), whereas haplotype (*t* = −4.93; df = 8,122; *p* = 0.0006) but not IQ was significant in controls (Supplementary Figure S5). Therefore, reduced FA in this regional juncture may negatively impact IQ in people with schizophrenia. There was a significant effect of age on FA-ROI in controls (*p* = 5.6 × 10^−5^, with a 5.9 × 10^−4^ unit reduction of FA for each year increase), with a trend effect in cases (*p* = 0.059, of −3.4 × 10^−4^ units/year). However, interactions between age, diagnosis, and haplotype were not significant.

## Discussion

Determining the link between the inheritance of specific genetic factors that influence risk of developing mental illness and their effects on neuroanatomy or brain function is pivotal to understanding the neurobiological mechanisms underlying the development of mental illness. This study shows that a haplotype in *ST8SIA2*, previously reported to be “protective”^12^, influences white matter microstructure in a regional juncture of association and projection fibers in the right parietal lobe. Furthermore, reduced FA in this region is associated with reduced IQ in schizophrenia patients, a core component of the schizophrenia phenotype^43^. This study provides an important link between human disease-associated genetic variants in *ST8SIA2* and specific brain deficits, providing further compelling evidence for involvement of this replicable^12,13,15,45,46^ and functionally relevant^20,30,47^ candidate gene in the pathophysiology of schizophrenia. To our knowledge, this is the first investigation of the differential effects of specific *ST8SIA2* genetic variants on brain white matter microstructure in patients with schizophrenia.

In the current study, we focused our analysis on two specific common haplotypes that were previously defined in independent cohorts with bipolar disorder or schizophrenia as “risk” (i.e., over-represented in case subjects compared to controls), or “protective” (i.e., over-represented in control subjects compared to cases).^12^ In a population, each individual carries two haplotypes from a pool of many possible haplotypes present in the population, and each haplotype block—typically defined by common SNP variants—can carry additional rare variation, which is in incomplete linkage disequilibrium with the tagging SNPs, making that haplotype unique. While the specific genetic variants affecting *ST8SIA2* gene function in schizophrenia are largely unknown (with the exception of the rare functional missense variant E141K (rs545681995)^13,16^), evolutionary theory suggests that both common and rare variation may contribute to phenotypic variability^48^, and this putative functional variation may have arisen on a spectrum of haplotypes^32^. Our analysis did not consider the identity of the second or “other” haplotype carried by each subject, which likely harbors a different spectrum of putative functional variation^32^. Hence, it is possible that the results we observe are influenced by the composition of “other” haplotypes within each group, or variants that are incompletely tagged by the variants examined, and this must be kept in mind with regards to interpretation of the findings. We note that the frequency of previously identified disease-associated *ST8SIA2* haplotypes^12^ did not differ with diagnosis in this independent cohort, which may be a consequence of reduced power of this neuroimaging sample for detecting significant genetic association.

We observed that the *ST8SIA2* “protective” haplotype is associated with an increase in FA in control subjects in a regional juncture of multiple fiber bundles, encompassing the SLF, corona radiata, and the posterior limb of the internal capsule. As such, modifications of white matter microstructure in this region may result in distributed network effects spanning multiple cortical and subcortical systems. Conversely, we observe an inverse effect of the “protective” haplotype in schizophrenia patients, where the haplotype is associated with reduced FA in the same region. The haplotype therefore appears to lose its “protective” effect in schizophrenia.

This apparent disparity may reflect the complex interplay between *ST8SIA2* and other genes that (individually or in concert) influence axonal fiber microstructure. Alternatively, these results may be explained in the context of a differential susceptibility model^49^, whereby a specific allele (or haplotype) of a gene can have either a beneficial or adverse effect, depending on the environmental condition in which it is measured. This differential susceptibility to environmental influence has been demonstrated previously for other genes and phenotypes^50^, and may also be relevant for *ST8SIA2*. However, as we did not formally test for specific gene × environment interactions in the current study, this explanation should be considered speculative.

We note that we did not observe an effect of the “risk” haplotype on white matter in this study. This was somewhat unexpected, given the observed significant effect of the protective haplotype—one might expect to see a concomitant reduction in FA in carriers of the risk haplotype. However, it may be that the specific genetic variants tagged by the risk haplotype are less penetrant in effects on white matter and thus would require larger samples to elucidate.

Previous research examining diagnostic group differences in FA has shown alterations affecting multiple cortical areas in schizophrenia^5^. In a previous analysis in the current cohort, individuals with schizophrenia had widespread reductions in FA compared to healthy controls^6^. Reduced FA in the SLF is not unique to chronic schizophrenia, but has also been found in individuals with first-episode psychosis^51–55^, bipolar disorder^56–60^, and autism^61–64^. The specific effect of *ST8SIA2* haplotype on this association fiber tract, in combination with data indicating that variation in *ST8SIA2* confers increased risk to (or protection against) each of these disorders^12,13,15,45^ is consistent with the link between *ST8SIA2* and the pathophysiology (or lack thereof) of a number of major psychiatric conditions.

The SLF is the largest association fiber bundle that mediates intrahemispheric corticocortical connections between frontal, temporal, and parietal lobes^65,66^. Although the exact role of the SLF is still largely unknown, the right bundle seems to be involved in visuospatial awareness^67^ and attention^68^, two functions that are impaired in patients with schizophrenia.

The corona radiata and posterior limb of the internal capsule contain white matter tracts connecting the cortex with subcortical regions including the thalamus (e.g., thalamocortical), brainstem (e.g., corticopontine), and spinal cord (e.g., corticospinal). Interestingly, the internal capsule has also shown reduced volume and disorganized pattern of fibers in mouse *st8sia2* knockout studies^30,31^.

Recent findings by Piras et al.^69^ further support the relevance of *ST8SIA2* to the SLF. They examined the relationship between PSA-NCAM (or polySia-NCAM) serum protein levels and human brain structure in healthy controls and schizophrenia patients with both structural MRI and DTI data. PSA-NCAM is formed by the addition of polysialic acid (polySia) chains on NCAM1 by polysialyltransferase enzymes (encoded by *ST8SIA2* and *ST8SIA4*), and NCAM1 is the major polySia carrier^70,71^. Intriguingly, they identified a significant positive relationship between peripheral PSA-NCAM protein levels and Brodmann area 46 (BA46) volume in healthy controls, and the inverse relationship in this same region of the dorsolateral prefrontal cortex in people with schizophrenia (i.e., reduced volume in BA46 with increased serum PSA-NCAM)^69^.

This is somewhat consistent with our findings involving the SLF in two ways. Firstly, the prefrontal region encompassing BA46 is one of the termini of the SLF—hence, one could expect to see alterations in gray matter in regions associated with affected white matter tracts^5^. Secondly, we also observed an inverse relationship in healthy controls as compared to people with schizophrenia with the protective haplotype. It must be noted, however, that, while PSA-NCAM formation will be influenced by the expression of the *ST8SIA2* gene and the activity of its resultant protein, PSA-NCAM protein levels will also reflect expression and activity of *ST8SIA4*, the alternative long-chain alpha-2,8 polysialic acid enzyme^71–73^, as well as the availability of glycosylatable NCAM1 isoforms (namely NCAM-140 and NCAM-180), which are differentially regulated over development^73^ (refs. ^20,71^). This indirect and confounded measure of ST8SIA2 may partly explain why Piras et al.^69^ did not find effects of serum PSA-NCAM protein levels on FA measures. The smaller sample size (*n* = 45 matched pairs) may also have contributed to their negative finding.

Together with previous work, the current study shows enticing data supporting the implication of *ST8SIA2* genetic variants in schizophrenia, and, notably, informs a potential mechanism for the protection against major mental illness in healthy individuals. Indeed, *ST8SIA2* should be considered a “plasticity gene”^49^ rather than a “risk gene” for two reasons. Firstly, it appears that the mechanism of association of the disease-associated haplotype may operate via the protective effect of the CGAC haplotype in resilient individuals (i.e., reducing risk to mental illness via modifications of white matter) rather than necessarily relating directly to the risk for specific psychopathological conditions (i.e., schizophrenia, autism, and bipolar disorder—as evidenced by altered allelic frequency when compared to a control population group^12,13,15,45,46^). Secondly, we know that *ST8SIA2* has direct effects in the developing vertebrate nervous system, as well as being involved in plasticity-related responses in adulthood^20^.

Finally, it must be noted that our analysis does not take into account the variation in the many other genes that also contribute risk of schizophrenia, which is a limitation of the current study, as it is with all gene-centric functional analyses. Furthermore, while FA is the most well-established and widely used neuroimaging measure reflecting axonal fiber density, diameter, and myelination^74^, it is a relatively indirect measure of white matter connectivity. Reductions in FA can also be a marker of intersecting fiber bundles, as DTI is prone to errors in resolving fiber crossings^75^. Therefore, independent validation of our findings utilizing alternative methods assessing fiber tract integrity is necessary. While future meta-analytic and mega-analytic replication studies are vital, this is currently challenging due to the difficulties in combining tractography findings across different sites, which will require the development of sophisticated harmonization protocols.

Recent data from mouse models suggest that the variability in FA observed in the human cohort is likely not an artifact of fiber crossings but a true reflection of the pathology in carriers of this haplotype. Mice lacking *st8sia2* have impairments in oligodendrocyte maturation, resulting in thinner myelin sheath and nerve fibers, which degenerate with age^76^, and downregulation of proteins involved in myelination processes (e.g., myelin basic protein, myelin proteolipid, and myelin-associated glycoprotein). Furthermore, in *st8sia2*-deficient mice, upregulation of proteins expressed from other genes implicated in the pathogenesis of bipolar disorder and schizophrenia (e.g., the Neurocan core protein, NCAN^77,78^) implies downstream dysregulation of additional disease-associated genes in the absence of *st8sia2*.

In conclusion, this study provides a link between a common haplotype in *ST8SIA2* and changes in white matter microstructure in resilience against or risk of schizophrenia, providing further compelling evidence for involvement of this “plasticity” gene in the pathophysiology (or lack thereof) of this complex, heterogeneous and polygenic disorder. Further studies into the effects of *ST8SIA2* genetic variants on white matter microstructure in schizophrenia and other psychiatric conditions—as well as resilient control populations—are warranted.

## Electronic supplementary material

supplementary material

Figure S1

Figure S2

Figure S3

Figure S4

Figure S5
