# Supplementary material for: Differential effect of disease-associated ST8SIA2 haplotype on cerebral white matter diffusion properties in schizophrenia and healthy controls
Source: Transl Psychiatry. 2018 Jan 22;8:21. doi: 10.1038/s41398-017-0052-z (PMC5802561; doi:10.1038/s41398-017-0052-z)
Supplement: Supplementary file 1 — supplementary material [file 41398_2017_52_MOESM1_ESM.docx]

**Supplementary Methods**

**Genotyping**

Genomic DNA extraction from peripheral blood was performed using a standard salt extraction method.^1^ DNA was diluted, aliquoted, and stored dried down at -80ºC until required. Each SNP (rs4586379, rs2035645, rs4777974 and rs3784735) was amplified using Taqman probes (assays on demand: C__27990275_10, C___9281327_10, C___9281306_10, C___9281224_20; Life Technologies, Mulgrave, Victoria, Australia), and allelic discrimination performed as per manufacturers’ instructions, using the 7900HT Real-Time PCR System, in conjunction with SDS v2.4. Results from multiple plates were combined using Taqman Genotyper software v1.3 (Life Technologies, Mulgrave, Victoria, Australia) with a no-amplification control annotated for each plate. Genotype clusters were manually inspected and edited to remove outliers. All genotypes were in Hardy-Weinberg equilibrium in control subjects (p values all > 0.24).

Linkage disequilibrium (LD) blocks were assessed using Haploview v4.2,^2^ and defined by Gabriel *et al* default parameters. Haplotypes were phased using the E-M algorithm implemented in PLINK,^3^ utilising the full ASRB cohort (p42 release) regardless of availability of DTI data (n=1314 subjects, of which 666 were cases and 636 were controls), plus genotypes from 1000 genomes project European populations (n=379 subjects from GBR and CEU cohorts)^4^ to enhance reliability of phased haplotypes. Subjects for whom the posterior probability for phased haplotype was <0.75 were excluded from further analysis.

Allele frequencies and correlations (r^2^) between genotypes were compared to those previously reported for European populations from the 1000 Genomes project,^4^ and were consistent with expectation (absolute frequency difference = 0.018±0.0083 (mean±SD); average r^2^ difference= 0.041 ± 0.027).

**Imputation**

Data from Infinium Human 610K BeadChips (Illumina, San Diego, CA, USA) was used, where available,^5^ to: 1) enable genotype-derive ethnicity to be determined; and 2) augment phased haplotypes where Taqman-derived haplotypes were deemed unreliable (posterior probability <0.75). Prior to imputation, SNPs with low minor allele frequency (<0.01), call rate (<95%), or deviation from Hardy–Weinberg equilibrium (p<1x10^-6^) were removed. Imputation was conducted using the ENIGMA imputation protocols (http://enigma.ini.usc.edu/), implementing MaCH^6^ and minimac.^7^ An additional 7 subjects were included from imputed data, for whom the average posterior probability of phased haplotypes was 0.843.

The concordance between imputed and Taqman-derived genotypes was high (96% rs3784735, and 91% for rs4777974), as was the concordance between SNPs directly genotyped via both microarray and Taqman (98% for rs4586379, and 99.9% for rs2035645).

**Multidimensional scaling and ethnicity estimation**

Genotype-derived ancestry was assessed by multi-dimensional scaling (MDS) analysis implemented in PLINK^3^, using the 1000 genomes reference panels^4^ and following ENIGMA protocols. Ancestry membership was determined from the first two principle components.

Genotype-derived ethnicity (n=333, 68% of sample) was compared to ethnicity based on grandparental country of birth (self-report), and was found to be consistent with self-report data for subjects where at least ¾ grandparental origins were known (n=296, 98.3% concordance). A small number of subjects with demographic grand-parental origin data coded as unknown (n=27, 8%) were determined via principle components to be European/mixed-European.

Based on the combination of self-report and genotype-derived ethnicity, the cohort was largely European (81.1%) or mixed-European (7.0%) with a small proportion of Asian (3.5%) and individuals of unknown ethnic origin (8.4%) [Supplementary Table S1].

**Multi-site image quality control**

The same Siemens Avanto 1.5 Tesla scanner model and image acquisition protocols were used at each of the five sites. Scanner calibration was conducted at each site using an identical Siemens phantom. We did not detect any site variation by collecting a scan from the same person travelling to each site. Detailed examination of differences in FA between scanning sites seems to relate primarily to power of individual samples rather than site-specific differences in FA.^8^ To remove any site-related variance, scanning-site was set as a nuisance factor in all statistical analyses. All images were manually inspected for artifacts. No scanner upgrades were performed at any site during the study lifetime.

**Fibre tracking**

Diffusion tensor streamlines were initiated for a control participant from the whole white matter segment (-seed option) and propagated using *streamtrack command* (DT_STREAM option), a deterministic white matter fiber tracking algorithm in MRtrix 0.2.12 (<http://www.nitrc.org/projects/mrtrix/>). Only the first 1000 streamlines passing though the cluster (*-include* option), with minimum length of 20 mm were kept. Visualization of fiber tracks passing through the cluster and superimposed on structural T1-weighted images was performed using TrackVis (Ruopeng Wang, Van J. Wedeen; TrackVis.org; Martinos Center for Biomedical Imaging, Massachusetts General Hospital).

**Post-hoc analyses**

Mean FA values across all voxels within the significant disease × haplotype interaction region were extracted for each subject. Post-hoc comparisons of mean FA values within each haplotype group were performed using independent non-parametric Mann-Whitney tests. To examine the relationship between FA within the region of interest and IQ, mean FA was used as a dependent variable in a regression analysis, which included IQ, age, gender and scanning site as predictors. A second model was tested which included the above as well as haplotype (0 or ≥1 copies) as a predictor, as well as an IQ × Haplotype interaction term, but the latter was not significant so it was removed from the final model. We also examined the effect of age on FA-ROI, due to its established relationship with both FA^9^ and *ST8SIA2* expression.^10,11^ This model included all variables as above, plus an age × haplotype interaction term. Case and control groups were examined separately, due to the large differences in mean IQ between case and control groups and their different distribution shape [Table 2; Figure S4]. Statistical analysis was conducted using SPSS software (Version 23, IBM Corporation, Armonk, NY).

**Supplementary Tables**

| **Ethnic Group** | **CON** | **SCZ** | **Total** |
| --- | --- | --- | --- |
| European | 140 (74.9%) | 256 (85.0%) | 396 (81.1%) |
| mixed-European | 7 (3.7%) | 27 (9.0%) | 34 (7.0%) |
| Asian | 9 (4.8%) | 8 (2.7%) | 17 (3.5%) |
| Unknown | 31 (16.6%) | 10 (3.3%) | 41 (8.4%) |
| Total | 187 (100%) | 301 (100%) | 488 (100%) |

**Table S1: Ethnic breakdown of cohort.** Ethnicity was derived from self-report (inferred from grandparental country of birth, with origins of at least 3 grandparents known) and, where available, genotype-derived using principle components analysis. Numbers of subjects in each ethnic group are given, with percentages in parentheses. Of the total, 112 control subjects (CON) and 220 schizophrenia cases (SCZ) had genotype-derived ethnicity to confirm self-report demographic data (68% of sample).

**Table S2: Demographics, clinical variables and risk haplotype (TTGA) carrier status**

|  | **Controls** | | | | **Patients** | | | | Controls vs Patients |
| --- | --- | --- | --- | --- | --- | --- | --- | --- | --- |
| **TTGA “risk” haplotype copies** | All (n=172) | 0 copies (n=64) | 1+ copies (n=108) | Carrier vs non-carrier | All (n=281) | 0 copies (n=115) | 1+ copies (n=166) | Carrier vs non-carrier | *X^2^* = 0.616 (p=0.432) |
| **Age** | 41.5  (18-64) | 40  (18-64) | 43.5  (19-62) | *U* = 3153 (p=0.337) | 38  (20-65) | 39  (20-64) | 37  (20-65) | *U* = 9044 (p=0.454) | *U* = 22208 (p=0.147) |
| **Sex** (Males; Females) | 87; 85 | 32; 32 | 55; 53 | *X*^2^ = 0.014 (p=0.906) | 196; 85 | 77; 38 | 119; 47 | *X^2^* = 0.720 (p=0.395) | ***X^2^* = 16.72**  **(p=4.3x10^-5^)** |
| **Handedness** | 90 | 95 | 90 | *U* = 3278 (p=0.55) | 100 | 100 | 100 | *U* = 9526 (p=0.975) | *U* = 21856  (p=0.066) |
| **WASI** | 119  (80-138) | 119.5  (80-137) | 119  (83-138) | *U* = 3388 (p=0.828) | 104  (58-133) | 103  (58-132) | 106  (69-133) | *U* = 9176 (p=0.582) | ***U* = 11645**  **(p=2.0x10^-20^)** |
| **Diagnostic**  (SCZ; SAD; SAB) |  | - | - | - |  | 98; 9; 8 | 141; 15; 10 | *X^2^* = 0.209 (p=0.90) | - |

DSM-IV diagnostics (SCZ, schizophrenia; SAD schizoaffective disorder of depressive type; SAB, schizoaffective disorder bipolar type); Handedness as measured by Edinburgh Handedness Scale^12^, a continuous laterality quotient scaled from -100 to +100, where negative values indicate propensity for left-handedness, and ±100 indicates unilaterality; WASI, Wechsler abbreviated scale of intelligence; “Carrier” refers to carrier of TTGA “risk” haplotype; If not otherwise specified, the values represent the median and the range is given in brackets; statistically significant differences (p<.05) are indicated in bold.

**Supplementary Figure Legends**


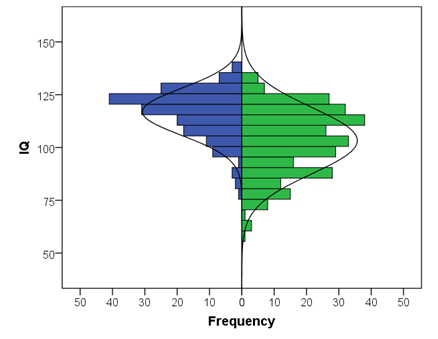


**Figure S1: Distribution of IQ scores across diagnostic groups.** Subjects with schizophrenia (green) had lower mean IQ than control subjects (blue), and a greater IQ range. For schizophrenia, mean=103.3, range(2SD)=71.9-134.7, interquartile range=25, skewness= -0.40, kurtosis= -0.53. For controls, mean=116.9, range(2SD)=94.6-139.2, interquartile range=15, skewness= -0.85, kurtosis= 0.71, respectively).

**
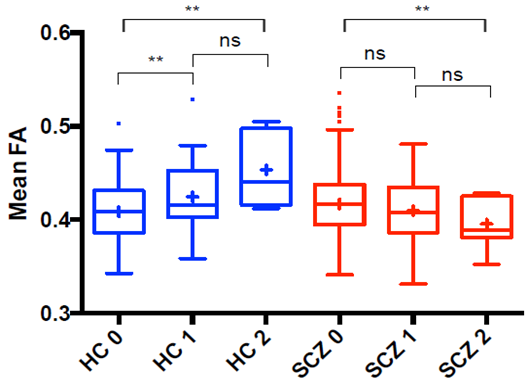
**

**Figure S2: Additive model showing differential effect of *ST8SIA2* protective haplotype on FA in healthy controls and subjects with schizophrenia (all subjects).** The distribution of mean FA values extracted from the significant cluster for healthy controls (HC, n=172) and subjects with schizophrenia (SCZ, n=281) carrying zero, one or two copies of the *ST8SIA2* protective haplotype. Whiskers represent a 1.5 interquartile range. Results of the post-hoc tests are indicated: * if *p* < 0.05; ** if *p* < 0.01.


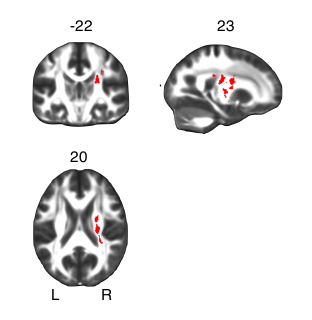


**Figure S3: Differential interaction effect of diagnosis and *ST8SIA2* protective haplotype on fractional anisotropy in the restricted sample of European/mixed-European subjects (n=401).** The red cluster (2401 voxels, p = .0052, FWER corrected) located on the right corona radiata, the superior longitudinal fasciculus and the posterior limb of the internal capsule represents voxels showing a significant interactive effect between *ST8SIA2* protective haplotype and diagnostic for schizophrenia on fractional anisotropy (FA). MNI coordinates are given on the top of each slice.

**
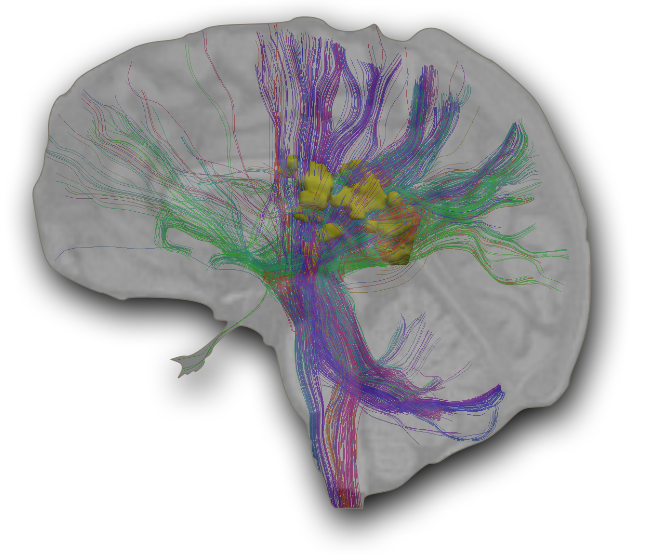
**

**Figure S4: The projection of the significant diagnosis x protective *ST8SIA2* haplotype interaction cluster** **from the restricted sample of European/mixed-European subjects (n=401).** Reconstructed fibres tracking through the yellow cluster (2401 voxels) descend from the right superior longitudinal fasciculus (SLF) and corona radiata (CR) through the posterior limb of the internal capsule (IC), extending into the cerebral peduncles (CP).

**
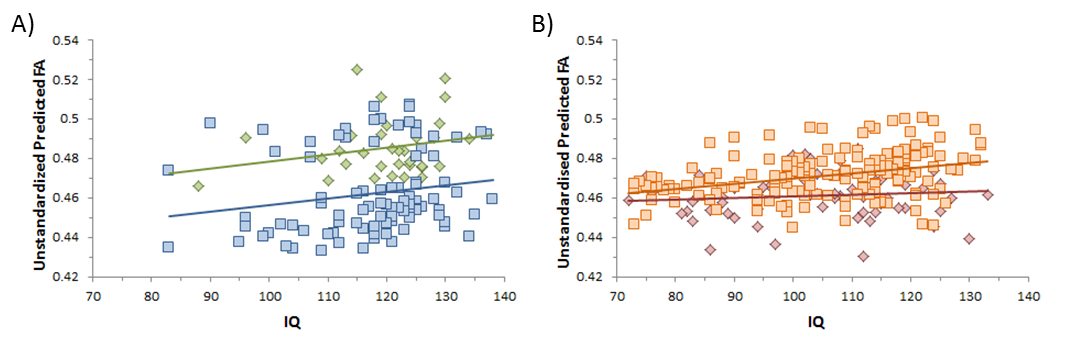
**

**Figure S5: Post-hoc analysis of relationship between mean FA within the region of interest, and both IQ and *ST8SIA2* haplotype in Caucasian subjects.** Mean FA values across all voxels within the significant disease x haplotype interaction region were extracted for each subject (FA-ROI), and used as a dependent variable in a regression analysis, which included IQ, haplotype (0 or ≥1 copies), age, gender and scanning site as predictors. Diamonds indicate subjects who carry the *ST8SIA2* protective haplotype, and squares indicate non-carriers. A) In control subjects, there was no significant correlation between FA-ROI and IQ (t=1.26; df=8,122; p=0.21), although the effect of *ST8SIA2* haplotype on FA-ROI was significant (t=-4.93; df=8,122; p=0.0006; R^2^=0.052 and 0.034 for non-carriers and carriers respectively). B) In schizophrenia patients, a positive correlation between FA-ROI and IQ (t=2.197; df=8,227; p=0.029) was observed, whereby FA-ROI was lower for subjects with lower IQ. A significant effect of haplotype was also observed (t=2.144; df=8,227; p=0.033; R^2^=0.12 and 0.011 for non-carriers and carriers respectively) whereby cases with lower FA-ROI carried the haplotype.

**Supplementary references**

1. Miller SA, Dykes DD, Polesky HF. A simple salting out procedure for extracting DNA from human nucleated cells. *Nucleic Acids Research* 1988; **16**(3)**:** 1215.

2. Barrett JC, Fry B, Maller J, Daly MJ. Haploview: analysis and visualization of LD and haplotype maps. *Bioinformatics* 2005; **21**(2)**:** 263-265.

3. Purcell S, Neale B, Todd-Brown K, Thomas L, Ferreira MA, Bender D *et al.* PLINK: a tool set for whole-genome association and population-based linkage analyses. *Am J Hum Genet* 2007; **81**(3)**:** 559-575.

4. 1000 Genomes Project Consortium, Auton A, Brooks LD, Durbin RM, Garrison EP, Kang HM *et al.* A global reference for human genetic variation. *Nature* 2015; **526**(7571)**:** 68-74.

5. Green MJ, Cairns MJ, Wu J, Dragovic M, Jablensky A, Tooney PA *et al.* Genome-wide supported variant MIR137 and severe negative symptoms predict membership of an impaired cognitive subtype of schizophrenia. *Mol Psychiatry* 2013; **18**(7)**:** 774-780.

6. Li Y, Willer CJ, Ding J, Scheet P, Abecasis GR. MaCH: using sequence and genotype data to estimate haplotypes and unobserved genotypes. *Genet Epidemiol* 2010; **34**(8)**:** 816-834.

7. Howie B, Fuchsberger C, Stephens M, Marchini J, Abecasis GR. Fast and accurate genotype imputation in genome-wide association studies through pre-phasing. *Nat Genet* 2012; **44**(8)**:** 955-959.

8. Klauser P, Baker ST, Cropley VL, Bousman C, Fornito A, Cocchi L *et al.* White Matter Disruptions in Schizophrenia Are Spatially Widespread and Topologically Converge on Brain Network Hubs. *Schizophrenia bulletin* 2016.

9. Cropley VL, Klauser P, Lenroot RK, Bruggemann J, Sundram S, Bousman C *et al.* Accelerated Gray and White Matter Deterioration With Age in Schizophrenia. *The American journal of psychiatry* 2016**:** appiajp201616050610.

10. Finne J, Finne U, Deagostini-Bazin H, Goridis C. Occurrence of alpha 2-8 linked polysialosyl units in a neural cell adhesion molecule. *Biochem Biophys Res Commun* 1983; **112**(2)**:** 482-487.

11. Eckhardt M, Muhlenhoff M, Bethe A, Koopman J, Frosch M, Gerardy-Schahn R. Molecular characterization of eukaryotic polysialyltransferase-1. *Nature* 1995; **373**(6516)**:** 715-718.

12. Oldfield RC. The assessment and analysis of handedness: the Edinburgh inventory. *Neuropsychologia* 1971; **9**(1)**:** 97-113.
