# Supplementary figures and images for: Differential effect of disease-associated ST8SIA2 haplotype on cerebral white matter diffusion properties in schizophrenia and healthy controls
Source: Transl Psychiatry. 2018 Jan 22;8:21. doi: 10.1038/s41398-017-0052-z (PMC5802561; doi:10.1038/s41398-017-0052-z)

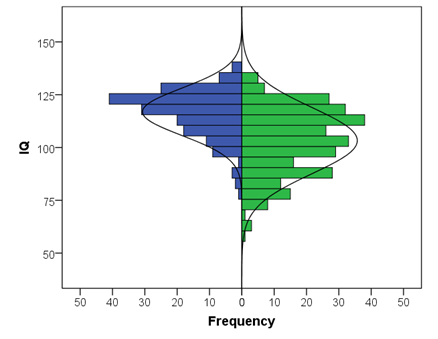

Supplement: Supplementary file 2 — Figure S1 [file 41398_2017_52_MOESM2_ESM.tif]

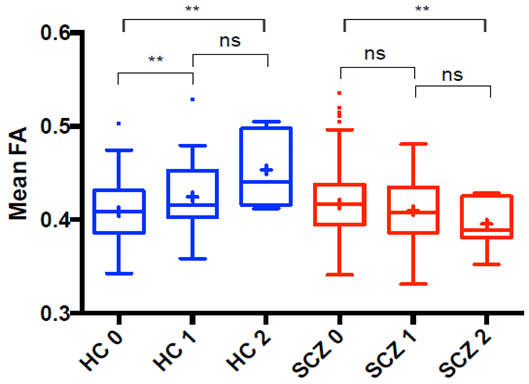

Supplement: Supplementary file 3 — Figure S2 [file 41398_2017_52_MOESM3_ESM.tif]

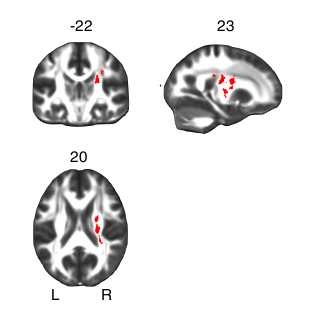

Supplement: Supplementary file 4 — Figure S3 [file 41398_2017_52_MOESM4_ESM.tif]

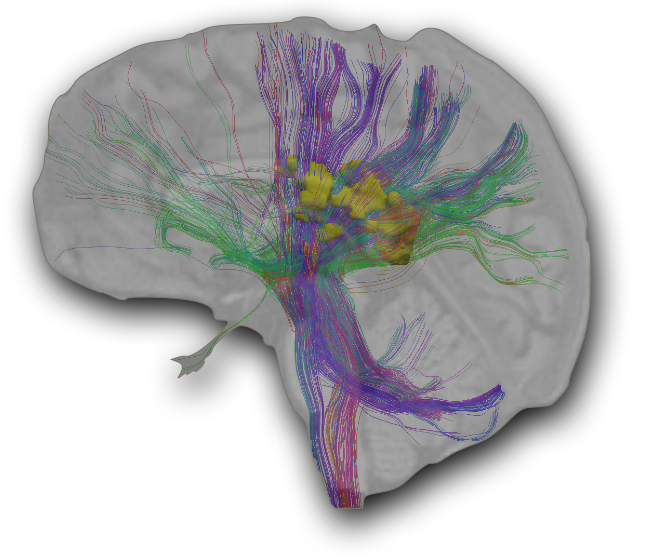

Supplement: Supplementary file 5 — Figure S4 [file 41398_2017_52_MOESM5_ESM.tif]

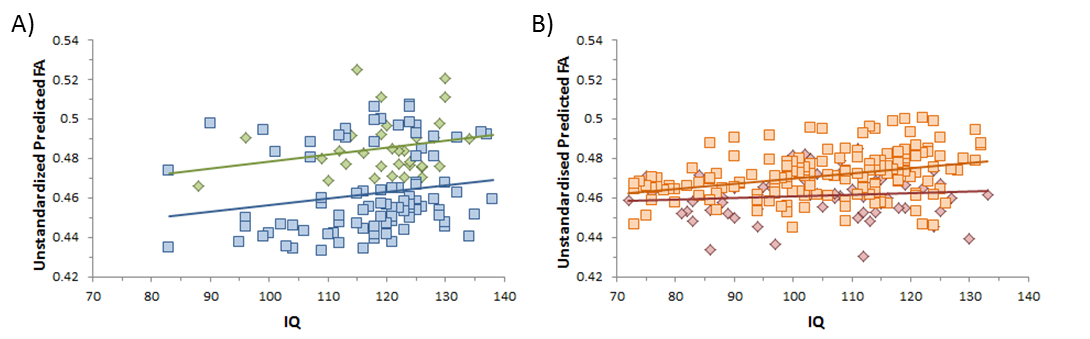

Supplement: Supplementary file 6 — Figure S5 [file 41398_2017_52_MOESM6_ESM.tif]
